# Activation of transient receptor potential vanilloid 4 protects articular cartilage against inflammatory responses via CaMKK/AMPK/NF-κB signaling pathway

**DOI:** 10.1038/s41598-021-94938-3

**Published:** 2021-07-30

**Authors:** Kyosuke Hattori, Nobunori Takahashi, Kenya Terabe, Yoshifumi Ohashi, Kenji Kishimoto, Yutaka Yokota, Mochihito Suzuki, Toshihisa Kojima, Shiro Imagama

**Affiliations:** Orthopaedic Surgery, Nagoya University Graduate School of Medicine, Nagoya, Aichi 446-8560 Japan

**Keywords:** Biochemistry, Molecular biology, Rheumatology

## Abstract

Transient receptor potential vanilloid 4 (TRPV4) plays an important role in chondrocytes via Ca^2+^ signaling. However, its role in the progression of osteoarthritis is unclear. This study aimed to evaluate the effects of TRPV4 activation on articular cartilage and chondrocytes stimulated with interleukin (IL)-1β. Bovine and human articular chondrocytes were stimulated with various agents, including IL-1β, GSK1016790A (GSK101; a TRPV4 agonist), Compound C (an AMP-activated protein kinase (AMPK) inhibitor), and STO-609 (a calmodulin-dependent protein kinase kinase (CaMKK) inhibitor), and were processed for Western blot analysis and real-time PCR. The dimethylmethylene blue (DMMB) assay and Safranin O staining were also performed. GSK101 reversed the IL-1β-induced increase in expression of matrix metalloproteinase (MMP)-13 and decrease in expression of aggrecan. GSK101 also decreased proteoglycan release in the DMMB assay and retained Safranin O staining of articular cartilage tissue. Furthermore, GSK101 increased AMPK phosphorylation and decreased IL-1β-induced nuclear factor kappa B (NF-κB) phosphorylation. Compound C and STO-609 reversed the suppressive effects of GSK101 on NF-κB activation and MMP-13 expression. In conclusion, TRPV4 activation had chondroprotective effects on articular cartilage stimulated with IL-1β by activating CaMKK/AMPK and suppressing the NF-κB pathway. TRPV4 activators may offer a promising therapeutic option for preventing the progression of osteoarthritis.

## Introduction

Transient receptor potential vanilloid 4 (TRPV4), an osmotically active ion channel associated with Ca^2+^ intake, plays an important role in mechano-transduction pathways of chondrocytes via Ca^2+^ signaling^1–3^. However, the role of TRPV4 in the progression of osteoarthritis (OA) is controversial. For instance, previous studies have shown that TRPV4 activation induced both catabolic and anabolic responses in chondrocytes in vitro^3–5^. Similarly, inconsistent results have been reported in TRPV4-knockout mice in vivo, with one study reporting progression of OA and another reporting a reduction of OA in these mice^6,7^.

We previously reported that TRPV4 stimulated with GSK101 plays a role in chondrogenesis by inducing the expression of chondrogenic markers including sex-determining region Y-box transcription factor (SOX9) and aggrecan (AGC)^8^, whereas signaling pathways of up-regulation of SOX9 and AGC via activation of TRPV4 was not well shown. It has been reported that Ca^2+^ intake activates AMP-activated protein kinase (AMPK), an evolutionarily conserved fuel and stress-sensing enzyme that can be activated by calmodulin-dependent protein kinase kinase-2 (CAMKK2) and that AMPK activation suppresses matrix degradation responses to interleukin (IL-)1β in chondrocytes^9,10^. Consistent with this, some drugs have been reported to attenuate cartilage degeneration by activating AMPK^11–14^. Although regulation of the CaMKK/AMPK/NF-κB signaling pathway inhibits inflammation, which plays a role in modern chronic diseases such as diabetes and cancer^15^, the role of this pathway in articular cartilage degradation and OA progression is unknown.

Based on the mechanistic findings discussed above, we hypothesized that TRPV4 may have a chondroprotective effect against arthritis caused by IL-1β stimulation. To test this, the present study aimed to determine whether TRPV4 activation in chondrocytes protects articular cartilage from degradation and inhibits the progression of OA via the CaMKK/AMPK/NF-κB signaling pathway.

## Results

### Determination of appropriate GSK101 concentration to inhibit IL-1β-induced cartilage degradation

**Figure 1:**
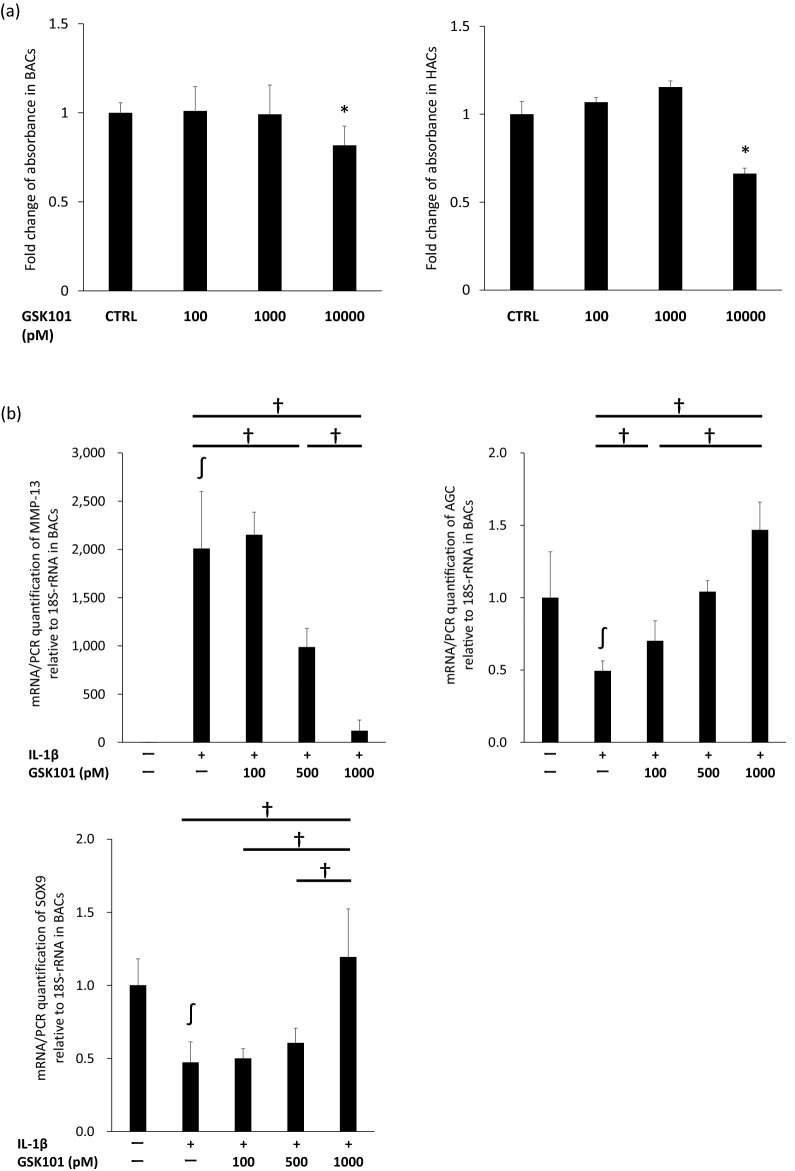
Optimization of GSK101 concentration. The optimal concentration of GSK101 for experiments was determined to be 1000 pM based on 48-h MTS assays in BACs and HACs, and real-time PCR-determined expression levels of MMP-13, aggrecan and SOX9 12 h after treatment in BACs. Experiments were repeated three times. (**a**) In the MTS assay, GSK101 was not cytotoxic both to BACs and HACs at concentrations of up to 1000 pM, but was cytotoxic at 10,000 pM. (**b**) In the real-time PCR in BACs, GSK101 significantly reversed the IL-1β-induced increase in expression of MMP-13 mRNA and decrease in expression of AGC and SOX9 mRNA in a dose-dependent manner. *p < 0.05 compared to untreated control, ∫*p* < 0.05 compared to untreated control, †*p* < 0.05 using one-way ANOVA with Tukey’s test. BAC: bovine articular cell; CTRL: control; HAC: human articular cell.

GSK101 was used as a specific TRPV4 agonist to explore the effects of TRPV4 activation on the pro-catabolic phenotype of activated chondrocytes. In an MTS assay, GSK101 was not cytotoxic to bovine articular chondrocytes (BACs) and human articular chondrocytes (HACs) at concentrations of up to 1000 pM, but was cytotoxic at 10,000 pM (Fig. 1a). In BACs, GSK101 significantly reversed the IL-1β-induced increase in expression of matrix metalloproteinase (MMP-)13 mRNA and decrease in expression of AGC and SOX9 mRNA in a dose-dependent manner (Fig. 1b). Since GSK101 was most effective at a concentration of 1000 pM, this concentration was used for subsequent experiments.

### TRPV4 activation inhibits IL-1β-induced cartilage degradation

**Figure 2:**
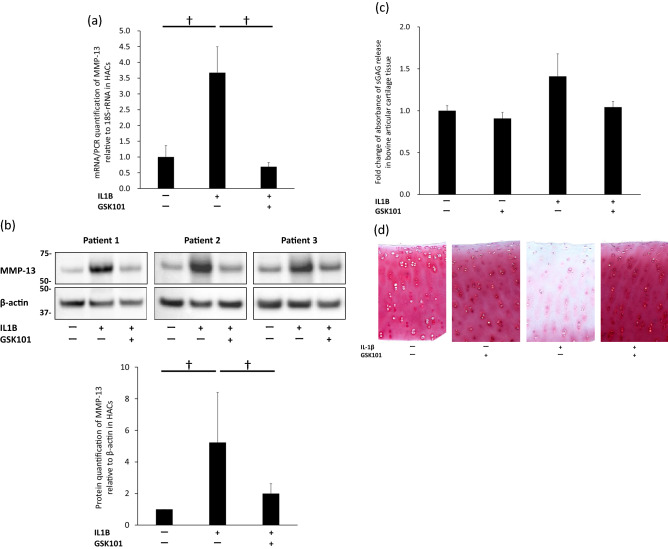
Reduction of IL-1β-induced cartilage damage by GSK101. (**a**) Real-time PCR of HAC lysates to examine the expression of MMP-13 after 12 h of treatment with 1000 pM GSK101, and (**b**) Western blot analysis of HAC lysates to examine the expression of MMP-13 after 48 h of treatment with 1000 pM GSK101. Experiments were repeated three times. Each group of the membrane associated with MMP-13 and the membrane associated with β-actin were different while their protein samples were the same. (**a**), (**b**) showed that GSK101 significantly reduced the expression of MMP-13 induced by IL-1β stimulation in HACs. Next, we performed DMMB assay on day 3 of explant culture and Safranin O/Fast Green staining on day 7 of explant culture of bovine articular cartilage tissue. Three replicates were used in each group. (**c**) In the DMMB assay, stimulating tissues with IL-1β significantly increased the elution of sGAG into the medium compared to the untreated control, while co-treatment with GSK101 significantly suppressed the IL-1β-induced release of sGAG. (**d**) In Safranin O/Fast Green staining of tissues after 7 days of IL-1β stimulation, similar tendency among three replicates was observed that substantial amounts of proteoglycans were lost from the explants and that these effects were almost completely rescued by co-treatment with GSK101. †*p* < 0.05 using one-way ANOVA with Tukey’s test. DMMB: dimethylmethylene blue; HAC: human articular cell; sGAG: sulfated glycosaminoglycan.

When examined by real-time PCR and Western blot analysis, GSK101 at 1000 pM significantly reduced the expression of MMP13 induced by IL-1β stimulation in HACs (Fig. 2a,b). To determine whether the addition of GSK101 can block proteoglycan release from bovine articular cartilage explants treated with IL-1β ex vivo, a dimethylmethylene blue (DMMB) colorimetric assay of sulfated glycosaminoglycan (sGAG) release after 3 days of IL-1β stimulation and Safranin O/Fast Green staining of bovine articular cartilage explant cores after 7 days of IL-1β stimulation were performed. Three replicates were used in each group both in the DMMB assay and Safranin O/Fast Green staining. In the DMMB assay, stimulating full-thickness 4-mm cores of cartilage explants with IL-1β significantly increased the elution of sGAG into the medium compared to the untreated control. Co-treatment with GSK101 significantly suppressed the IL-1β-induced release of sGAG (Fig. 2c). In Safranin O/Fast Green staining of bovine articular cartilage explant cores after 7 days of IL-1β stimulation, similar tendency among three replicates was observed that substantial amounts of proteoglycans were lost from the explants and that these effects were almost completely rescued by co-treatment with GSK101 (Fig. 2d).

### Pathways involved in chondroprotective effect of TRPV4 activation

**Figure 3:**
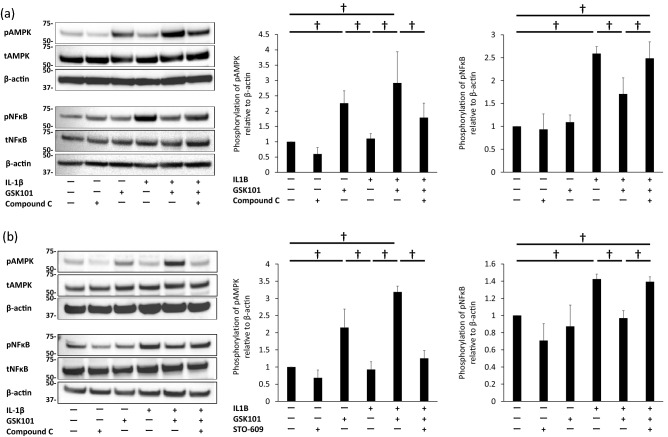
Inhibition of NF-κB phosphorylation by activation of the CaMKK/AMPK pathway. Levels of pAMPK and pNF-κB in BACs treated with (**a**) IL-1β, GSK101, and Compound C (25 µM), and (**b**) IL-1β, GSK101, and STO-609 (5 µM) for 30 min, as assessed by Western blot. Experiments were repeated three times. Each group of pNFκB, tNFκB and β-actin was derived from the same membrane. Each group of pAMPK, tAMPK and β-actin was derived from the same membrane. The membrane associated with NFκB and the membrane associated with AMPK were different while their protein samples were the same. (**a**), (**b**) showed that stimulation with IL-1β for 30 min increased levels of pNF-κB, and GSK101 treatment for 30 min enhanced levels of pAMPK, compared with untreated control samples. (**a**) showed that pre-treatment with compound C significantly suppressed phosphorylation of AMPK and increased phosphorylation of NF-κB compared to cells treated with IL-1β and GSK101. (**b)** showed that pre-treatment with STO-609 for 1 h significantly suppressed phosphorylation of AMPK observed with the combination of GSK101 and IL-1β, and also countered the suppressive effect of GSK101 on IL-1β-induced NF-κB phosphorylation. ∫*p* < 0.05 compared to untreated control; †*p* < 0.05 using one-way ANOVA with Tukey’s test. AMPK: AMP-activated protein kinase; BAC: bovine articular cell; NFκB: nuclear factor kappa B; p-: phosphor-; t: total-.

To identify signaling pathways which may be involved in the chondroprotective effect of GSK101, BACs were stimulated with IL-1β in the absence or presence of GSK101. Western blot and band densitometry analyses revealed that stimulation with IL-1β for 30 min increased the levels of phospho-(p)NF-κB (*p* < 0.001 in Fig. 3a and *p* = 0.0026 in Fig. 3b,respectively), and GSK101 treatment for 30 min enhanced the levels of pAMPK (*p* = 0.0028 in Fig. 3a and *p* = 0.0031 in Fig. 3b, respectively), compared with untreated control samples. Co-treatment with GSK101 significantly suppressed IL-1β-induced NF-κB phosphorylation (*p* = 0.0147 in Fig. 3a and *p* = 0.0164 in Fig. 3b, respectively).

Compound C, a chemical inhibitor of AMPK phosphorylation, was used to determine whether the phosphorylation of AMPK is involved in the GSK101-mediated suppression of IL-1β-induced NF-κB phosphorylation (Fig. 3a). Pre-treatment with compound C significantly suppressed the phosphorylation of AMPK (*p* = 0.006) and increased the phosphorylation of NF-κB (*p* = 0.033) compared to cells treated with IL-1β and GSK101.

STO-609, an inhibitor of CaMKK activation, was used to determine whether CaMKK activation cross-talked with the AMPK/NF-κB pathway (Fig. 3b). Pre-treatment with STO-609 for 1 h significantly suppressed the phosphorylation of AMPK observed with the combination of GSK101 and IL-1β (*p* < 0.001), and also countered the suppressive effect of GSK101 on IL-1β-induced NF-κB phosphorylation (*p* = 0.026).

These results collectively suggest that TRPV4 activation suppresses IL-1β-induced NF-κB activation by activating the CaMKK/AMPK pathway.

### TRPV4-mediated suppression of IL-1β-induced cartilage degradation via the CaMKK/AMPK/NF-κB pathway

As discussed above, the TRPV4/CaMKK/AMPK pathway is involved in suppressing IL-1β-induced NF-κB activation. Thus, we examined whether this mechanism was involved in the chondroprotective effect of TRPV4.

**Figure 4:**
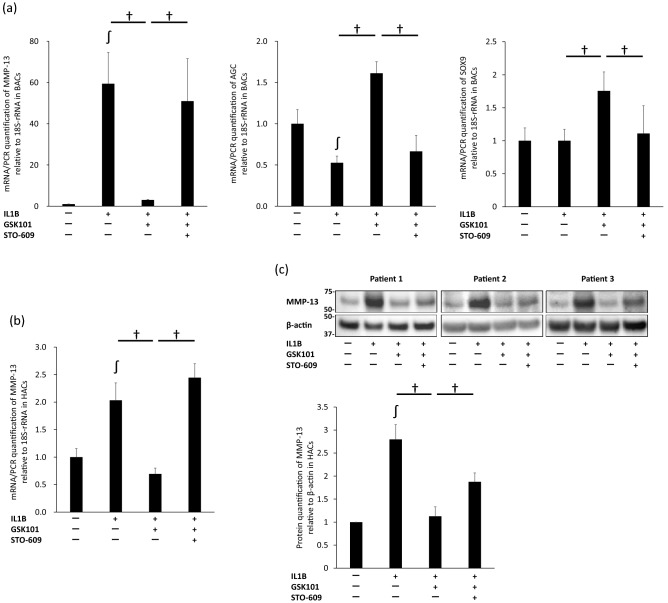
Inhibition of IL-1β-induced cartilage damage via the TRPV4/CaMKK pathway. (**a**) Real-time PCR analysis of relative expression of MMP-13, AGC and SOX9 in BACs treated with IL-1β, GSK101, and STO-609 for 12 h. Pre-treatment with STO-609 canceled the suppressive effect of GSK101 on IL-1β-induced up-regulation of MMP-13 and down-regulation of AGC mRNA. Pre-treatment with STO-609 also canceled the suppressive effect of GSK101 on up-regulation of SOX9 mRNA. (**b**) Real-time PCR of relative expression of MMP-13 in HACs treated with IL-1β, GSK101, and STO-609 for 12 h, and (**c**) Western blot analysis of relative expression of MMP-13 in HACs treated with IL-1β, GSK101, and STO-609 for 48 h. (**b**), (**c**) showed that pre-treatment with STO-609 canceled the suppressive effect of GSK101 on IL-1β-induced up-regulation of MMP-13 mRNA and protein. Each experiment was repeated three times. Each group of the membrane associated with MMP-13 and the membrane associated with β-actin were different while their protein samples were the same. ∫*p* < 0.05 compared to untreated control; †*p* < 0.05 using one-way ANOVA with Tukey’s test. BAC: bovine articular cell; CaMKK: calmodulin-dependent protein kinase kinase; HAC: human articular cell.

Both MMP-13 mRNA expression in BACs and HACs (n = 3, respectively) and MMP-13 protein expression in HACs (since the anti-MMP-13 antibody did not react with bovine MMP-13) under various conditions were examined. In BACs (Fig. 4a), pre-treatment with STO-609 canceled the suppressive effect of GSK101 on IL-1β-induced up-regulation of MMP-13 mRNA and down-regulation of AGC mRNA (*p* < 0.001 and *p* < 0.001, respectively). Pre-treatment with STO-609 also canceled the suppressive effect of GSK101 on up-regulation of SOX9 mRNA in BACs. Effects of STO-609 were also observed on MMP-13 mRNA and protein levels in HACs (*p* < 0.001 and *p* = 0.044, respectively; Fig. 4b,c). These results suggest that activation of CaMKK by TRPV4 is involved in the chondroprotective effect of TRPV4 against IL-1β-induced cartilage degradation.

## Discussion

Although recent studies have found that drugs such as metformin and protectin DX attenuate cartilage damage via the AMPK/NF-κB pathway^12–14^, the underlying mechanism was unclear. In addition, while the CaMKK/AMPK pathway has been reported to play an important role in myocytes and in circumventricular organs^16,17^, the role of this pathway in chondrocytes was unknown. The present study is the first to demonstrate that TRPV4 activation inhibits NF-κB phosphorylation by activating the CaMKK/AMPK pathway in chondrocytes in the presence of IL-1β, resulting in a chondroprotective effect.

Jeon et al. previously reported that exercise and contraction induce AMPK activation and inhibit NF-κB activation by increasing the AMP/ATP ratio and/or through the Ca^2+^/CaMKK signaling pathway in the context of diabetes and cancer^15^. Moreover, while moderate cyclic tensile strain suppresses IL-1β-induced inflammatory responses in chondrocytes via the AMPK/NF-κB pathway by increasing the AMP/ATP ratio^18^, no study has reported on the role of the CaMKK/AMPK/NF-κB pathway in chondrocytes. We found that TRPV4 activation down-regulated the expression of MMP-13 by activating CaMKK and AMPK and inactivating NF-κB. TRPV4 activation also up-regulated the expression of cartilage phenotypic genes, including SOX9 and AGC. Given the role of IL-1β in OA progression by inducing catabolic responses and inhibiting anabolic responses^19–22^, our findings suggest that TRPV4 activation by mechanical stress or chemicals may protect articular cartilage from degeneration and OA progression induced by IL-1β.

The role of TRPV4 in OA has been controversial. In vitro, some studies reported that TRPV4 activation induces catabolic responses in chondrocytes (e.g., increasing the expression of a disintegrin and metalloproteinase domain-containing protein (ADAM)10 and apoptosis of chondrocytes)^4,5^, while others reported the anabolic effects of TRPV4 activation (e.g., increasing the expression of type 2 collagen and decreasing the expression of disintegrin and metalloproteinase with thrombospondin motifs-5)^3^. This inconsistency has been noted in vivo as well. For instance, while one study reported that male TRPV4 knockout mice exhibited early and severe development of age-related OA^11^, another reported that the knockout mice were protected from age-related OA, but not from OA caused by destabilization of the medial meniscus^7^. In the present study, we observed the anabolic effects of TRPV4 activation, as reflected in the increased expression of AGC and SOX9 and decreased expression of MMP-13, as well as the preservation of proteoglycans in articular cartilage tissue in the IL-1β stimulation model. Given the possibility that the effects of TRPV4 activation may differ by experimental model, further confirmatory studies will be needed.

The inconsistent effects of TRPV4 activation in chondrocytes discussed above may reflect differences in the mechanism of AMPK activation. Indeed, AMPK is known to be activated by various mechanisms, including canonical and non-canonical pathways. As we have shown here, in one non-canonical pathway, AMPK is activated via a Ca^2+^/CaMKK2-dependent mechanism. Another non-canonical pathway in which AMPK is activated by glucose starvation was recently reported by Li et al.^23^. In that study, endoplasmic reticulum-localized TRPVs channels and Ca^2+^ release were inhibited by fructose-1,6-bisphosphate (FBP)-unoccupied aldolase under low glucose conditions, subsequently leading to the formation, phosphorylation, and activation of an AXIN-LKB1-AMPK complex on the lysosomal membrane. Importantly, GSK101 inhibited AMPK activation under glucose starvation conditions due to an increase in local Ca^2+^ concentrations, suggesting that the concentration of the TRPV agonist may lead to differing results because high concentrations can induce a bulk, global increase in Ca^2+^ concentration via a CaMKK2-dependent mechanism^23^. These results suggest that the effect of TRPV4 activation may depend on various factors, for example, the type of cell, the degree of inflammation, and glucose conditions.

The mechanism underlying the inhibition of NF-κB phosphorylation by AMPK activation remains unclear. Previous studies have reported that activation of AMPK or sirtuin (SIRT)1 inhibited IL-1β-induced inflammatory responses by inhibiting NF-κB activation in chondrocytes^11,24,25^. The activation of peroxisome proliferator activated receptor γ coactivator (PGC)-1α and Forkhead box O (FOXO)3a by AMPK activation was also reported to inhibit NF-κB activation and inflammatory cytokine-induced catabolic responses in chondrocytes^26^. Given that AMPK activation was reported to induce PGC-1α activation directly or via SIRT1 activation in myocytes^16^, the AMPK/SIRT1/PGC-1α pathway may play a role in suppressing IL-1β-induced inflammatory responses by inhibiting NF-κB activation in chondrocytes as well. The link between AMPK activation and suppression of NF-κB may also involve changes in glucose metabolism. In this regard, we recently reported that aerobic respiration switched to glycolysis in IL-1β-stimulated chondrocytes, and that IL-1β reduced the phosphorylation of AMPK, which was rescued by a chemical glycolysis inhibitor^10^.

In conclusion, the activation of TRPV4 suppressed IL-1β-induced chondro-degenerative changes and inflammatory responses including up-regulation of MMP-13 expression and down-regulation of AGC and SOX9 in chondrocytes by activating CaMKK/AMPK and suppressing the activation of NF-κB. As it has been reported that the Ca^2+^/CaMKK signaling pathway induces AMPK activation and inhibition of NF-κB activation in some chronic inflammatory diseases^15^, TRPV4/ CaMKK/AMPK pathway played a key role against IL-1β-induced articular cartilage degradation and OA progression. Although the mechanism underlying the inhibition of NF-κB phosphorylation by AMPK activation remains unclear, it has been reported that some other signaling pathways including SIRT1, PGC-1α and FOXO3a, which are associated with AMPK, inhibits IL-1β-induced inflammatory responses by inhibiting NF-κB activation in chondrocytes^11,24–26^. Taken together, TRPV4 activators may offer a promising therapeutic option for preventing OA progression.

## Methods

### Cells and cell culture

BACs were isolated from full-thickness slices of the articular surface of metatarsophalangeal joints of young adult cows (aged 18–24 months) which were obtained from Nagoya City Central Wholesale Market in Japan with institutional approval. No live animals were used in this study. HACs were isolated from slices of knee joints of patients who underwent total knee arthroplasty with institutional IRB approval (Ethics Committee of the Nagoya University Graduate School of Medicine #2020-0146). A written informed consent was obtained from the participants. with the World Medical Association of Helsinki Ethical Principles for Medical Research Involving Human Subjects. Also, these tissues were obtained with no identifying information except age/sex. All methods were carried out in accordance with relevant guidelines and regulations. Slices of bovine and human articular cartilage were digested in 0.2% Pronase (≥ 70,000 proteolytic units/g dry weight, Catalog #: 537088; Merck, Germany) for 1 h at 37 °C and subsequently in 0.025% collagenase P (> 1.5 U/mg lyophilizate; Catalog #: 11213865001; Roche, Germany) overnight at 37 °C^27^. Isolated cells were cultured in Dulbecco’s Modified Eagle’s Medium (DMEM) low glucose medium with 4% fetal bovine serum (FBS) and 1% antibiotics at 37 °C in a 5% CO_2_ environment. After 48–72 h in culture, cells were passaged once (P1) and 1–2 × 10^5^ cells/cm^2^ were cultured on 6- or 12-well plates. After static incubation for 48–72 h in 4% FBS-containing medium, cells were cultured in serum-free medium for 12 h. Subsequently, cells were stimulated in the presence of various agents, including IL-1β (10 ng/ml), various concentrations of GSK101 (a selective TRPV4 agonist), Compound C (an AMPK inhibitor; 25 µM), and STO-609 (a CaMKK inhibitor; 5 µM), under serum-free conditions. Cells were collected after stimulation and processed for Western blot analysis and real-time PCR. As we used primary chondrocytes isolated from bovines and humans whose age, sex and conditions of articular cartilage were different, potential variability due to individual differences among reactivity of chondrocytes to IL-1β might occur in the data from Western blot analysis and real-time PCR.

### Cartilage explant cultures

Full-thickness 4-mm cores of bovine articular cartilage were cultured in 1.0 ml DMEM low glucose medium with 4% FBS for 24 h. The medium was then replaced, and tissues were incubated with IL-1β (10 ng/ml), with or without GSK101. On day 3 of culture, aliquots of medium were analyzed by the DMMB colorimetric assay to measure sGAG release. On day 7, the treated explants were fixed with 4% buffered paraformaldehyde overnight at 4 °C for histology; rinsed in 30% sucrose/PBS; and embedded in paraffin. Sections (8 µm) were prepared and stained with Safranin O for the detection of proteoglycans and counterstained with Fast Green^10^.

### Real-time PCR

Total RNA was extracted with the RNeasy Mini Kit (Qiagen, Germany). Reverse transcription (RT) was performed using the High Capacity cDNA Reverse Transcription Kit (Applied Biosystems, USA). Real time RT-PCR was carried out using a Light cycler System with FastStart Master SYBR Green PLUS (Roche, USA)^27^. Primers for matrix metalloproteinase (MMP)-13, AGC, SOX9, and 18S ribosomal RNA (18S rRNA) in bovine and human were synthesized by Sigma-Aldrich (USA). The following primers were used: bovine MMP-13, forward primer 5′-TCCAGTTTGCAGAGAGCTACCT-3′, reverse primer 5′-CCTGTCAATCACAGAGCTTGCT-3′; bovine AGC, forward primer 5′-AAATATCACTGAGGGTGAAGCCCG-3′, reverse primer 5′-ACTTCAGGGACAAACGTGAAAGGC-3′; bovine SOX9, forward primer 5′-CGACTCCCCACATTCCTCCTC-3′, reverse primer 5′-GGACCCTGAGATTGCCCAGAG-3′; bovine 18S rRNA, forward primer 5′-GTAACCCGTTGAACCCCATT-3′, reverse primer 5′-CCATCCAATCGGTAGTAGCG-3′; human MMP-13, forward primer 5′-CCAGTCTCCGAGGAGAAACA-3′, reverse primer 5′-AAAAACAGCTCCGCATCAAC-3′; and human 18S rRNA, forward primer 5′-CCGATTGGATGGTTTAGTGAG-3′, reverse primer 5′-AGTTCGACCGTCTTCTCAGC-3′.

### Western blot analysis

The protein expressions of MMP-13, AGC, AMPK, and NF-κB were evaluated by Western blot analysis using BAC and HAC lysates. Cells cultured on 6-well plates were trypsinized and pelleted by centrifugation. Total protein was extracted from cell pellets with Cell Lysis Buffer (Cell Signaling, USA) containing a protease and phosphatase inhibitor cocktail. Samples were separated by 10% SDS-PAGE under reducing conditions and transferred to a nitrocellulose membrane. Antibodies against MMP-13 (18165-1-AP, Proteintech Group), AGC (ab3778, abcam), pAMPK (2535, Cell Signaling), AMPK (5831, Cell Signaling), pNF-κB (3033, Cell Signaling), NF-κB (8242, Cell Signaling), and beta-actin (4970, Cell Signaling) were used. Band intensities were captured with a digital image scanner and quantified using densitometry software (CS Analyzer 3.0; ATTO, Tokyo, Japan).

### Statistical analysis

Values are expressed as mean ± standard deviation (SD). One-way ANOVA with Tukey’s test was performed for comparisons. Statistical significance was defined as *p* < 0.05. All analyses were performed with BellCurve for Excel version 3.21.

## Supplementary Information

Supplementary Information.

## Data Availability

All data generated or analyzed during this study are includes in this published article.
