# Supplementary material for: Activation of transient receptor potential vanilloid 4 protects articular cartilage against inflammatory responses via CaMKK/AMPK/NF-κB signaling pathway
Source: Sci Rep. 2021 Jul 30;11:15508. doi: 10.1038/s41598-021-94938-3 (PMC8324869; doi:10.1038/s41598-021-94938-3)
Supplement: Supplementary file 1 — Supplementary Information. [file 41598_2021_94938_MOESM1_ESM.pdf]

Supplementary data 1

**Expression of MMP-13 in HACs in the Figure 2b**

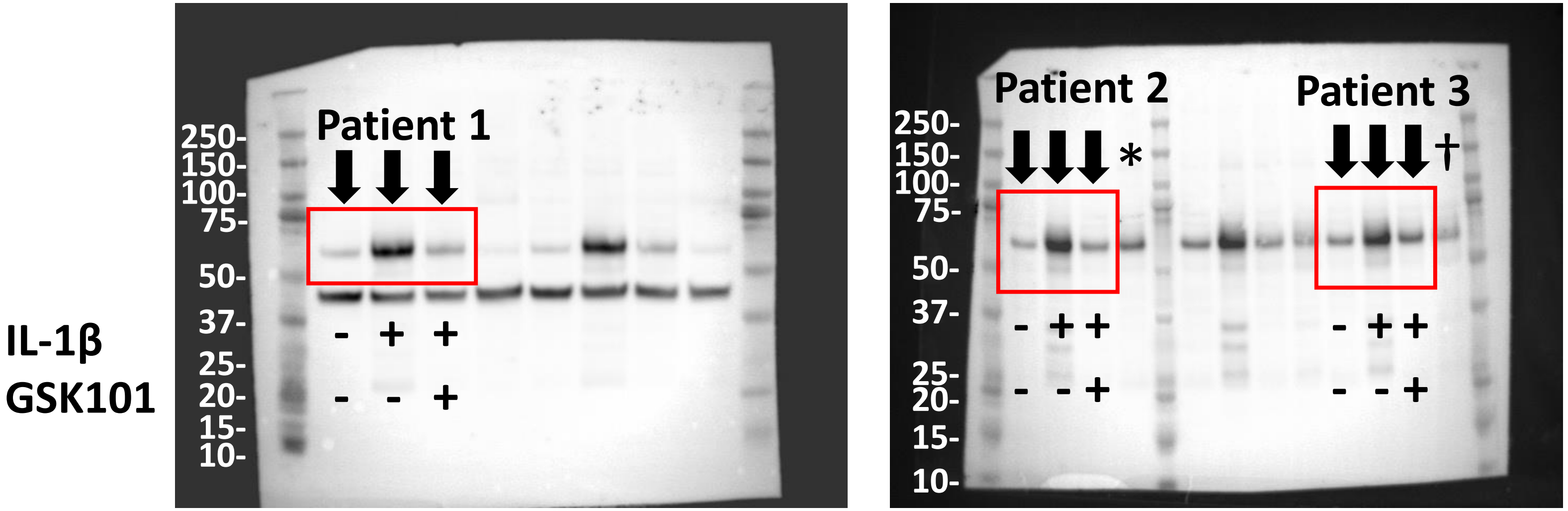


**Expression of β-actin in HACs in the Figure 2b**

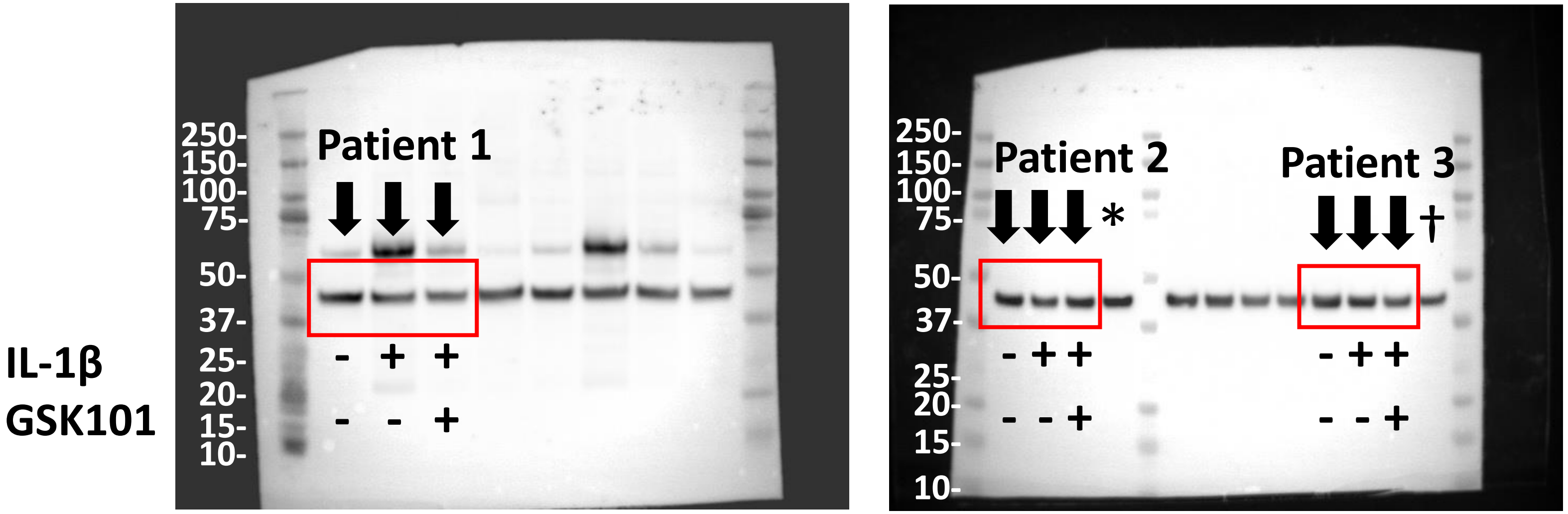

# Supplementary data 2

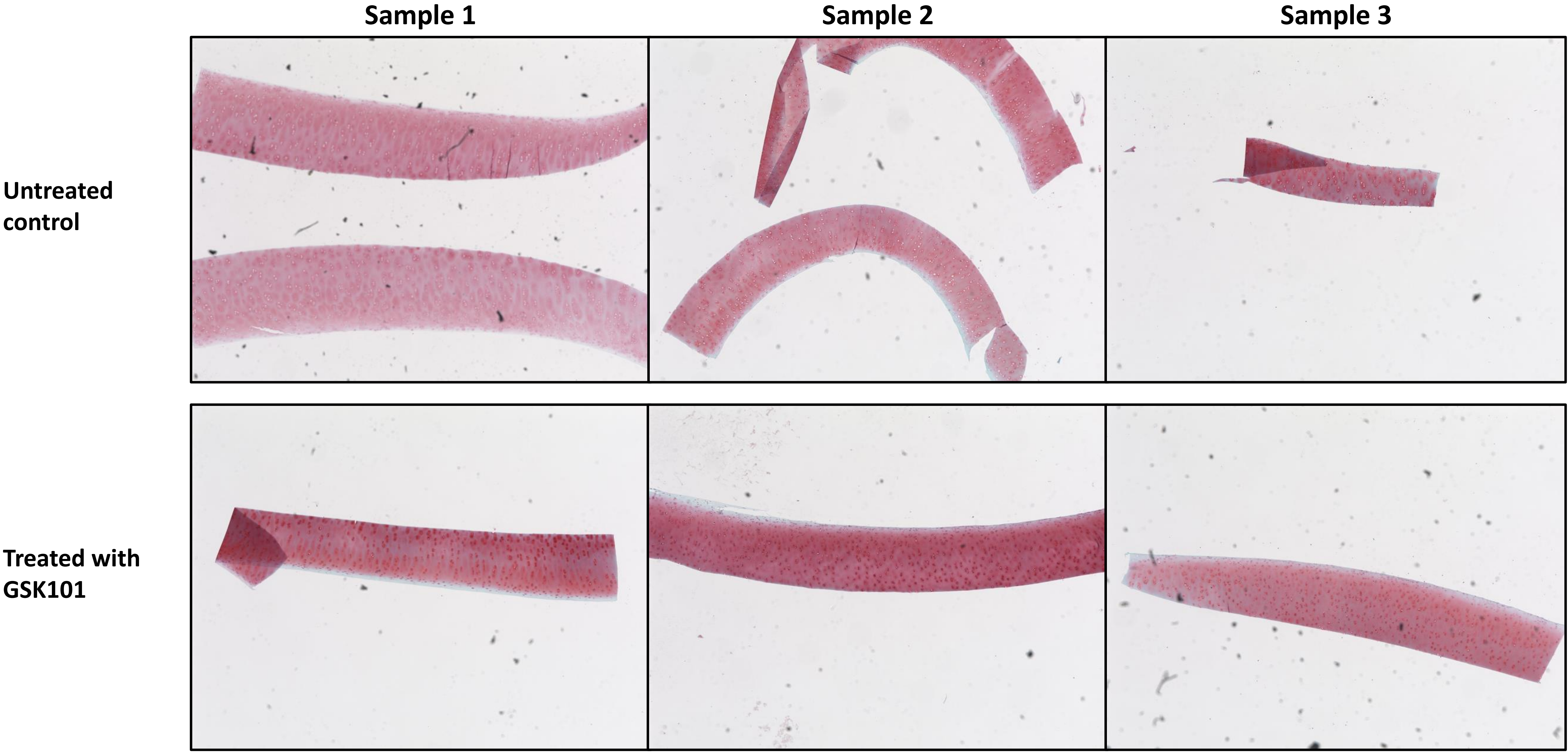

# Supplementary data 2 (continued)

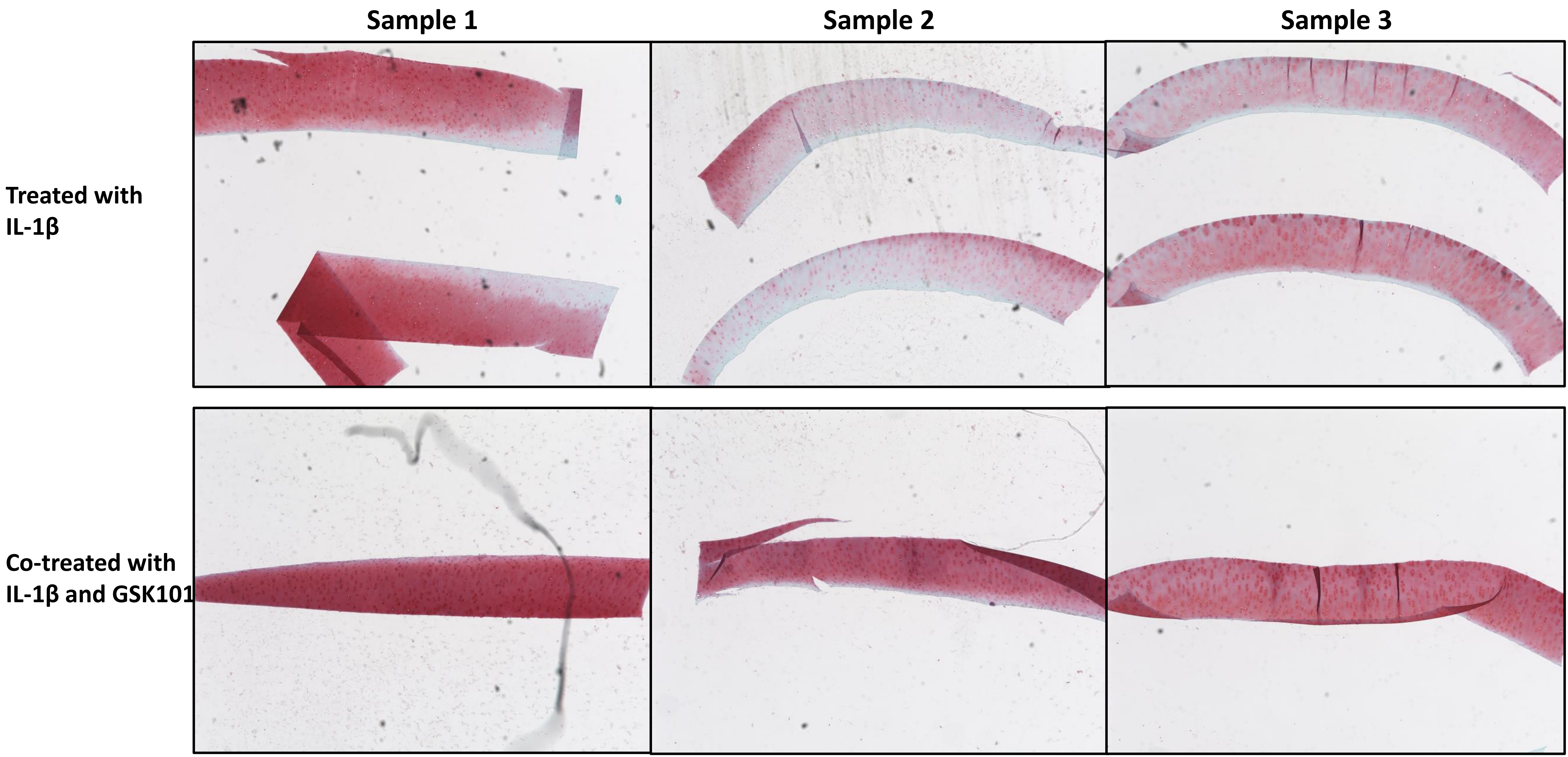

# Supplementary data 3(a)

**Expression of pAMPK, tAMPK and β-actin in BACs in the Figure 3a**

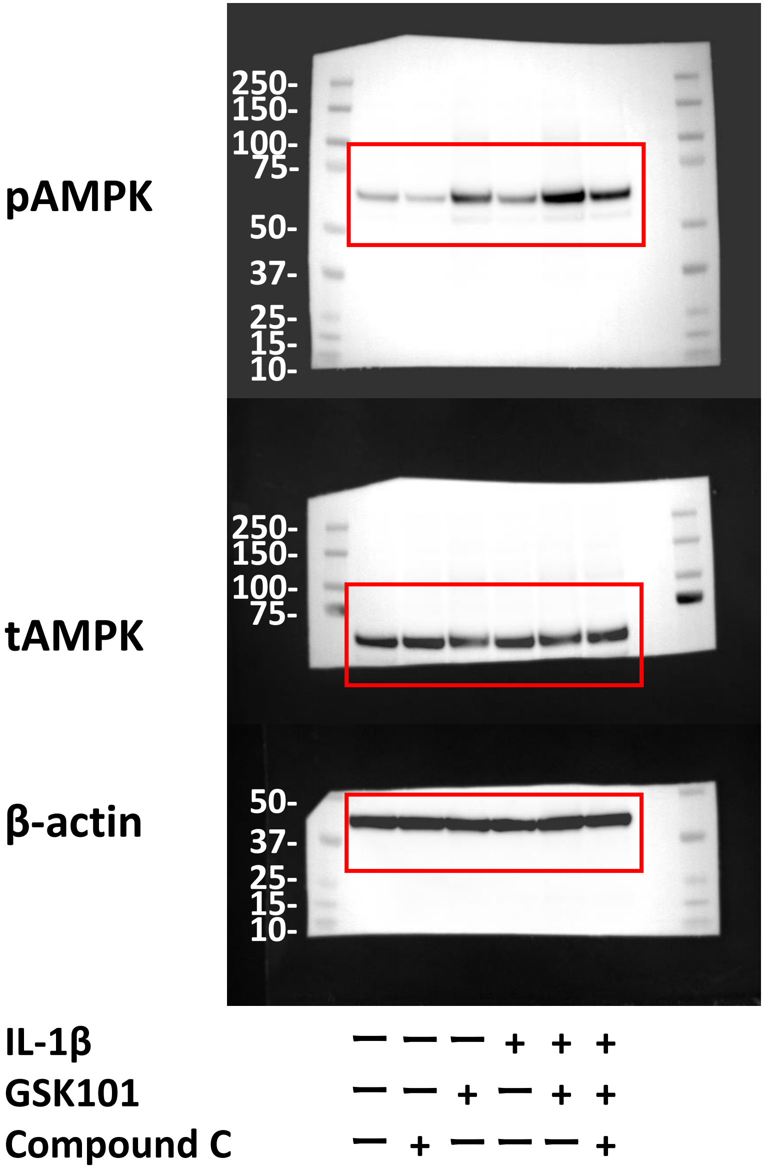


**Expression of pNFκB, tNFκB and β-actin in BACs in the Figure 3a**

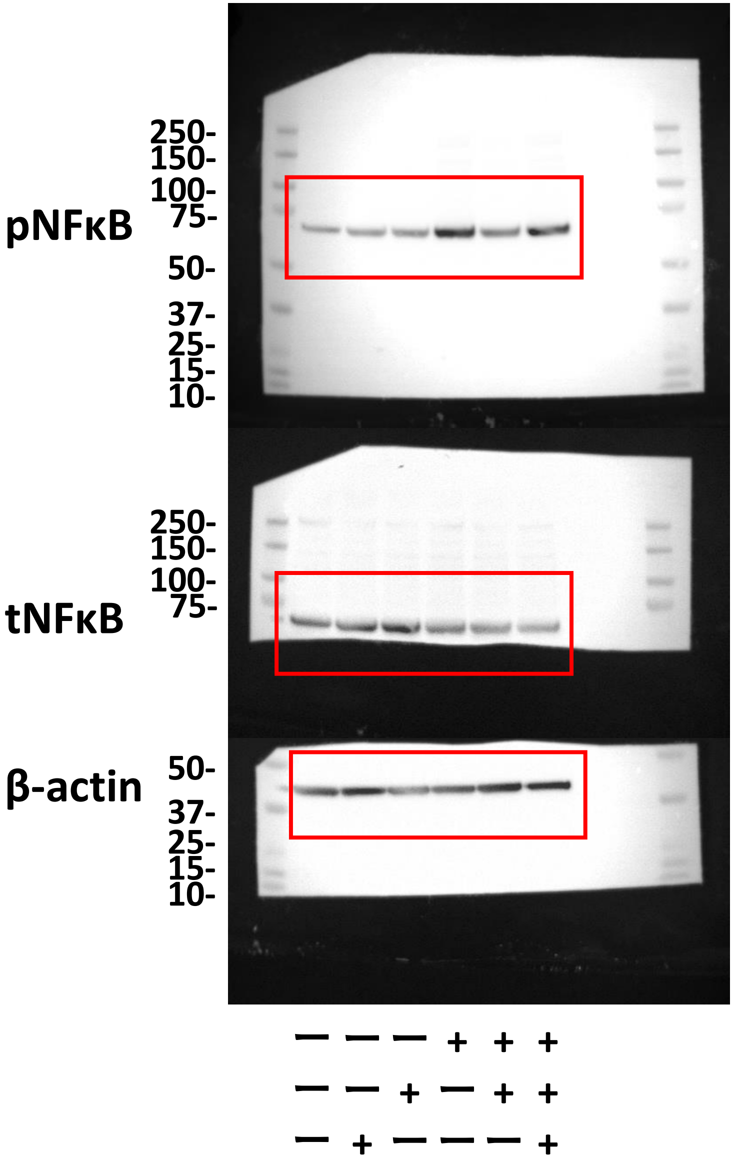

# Supplementary data 3(b)

**Expression of pAMPK, tAMPK and β-actin in BACs in the Figure 3b**

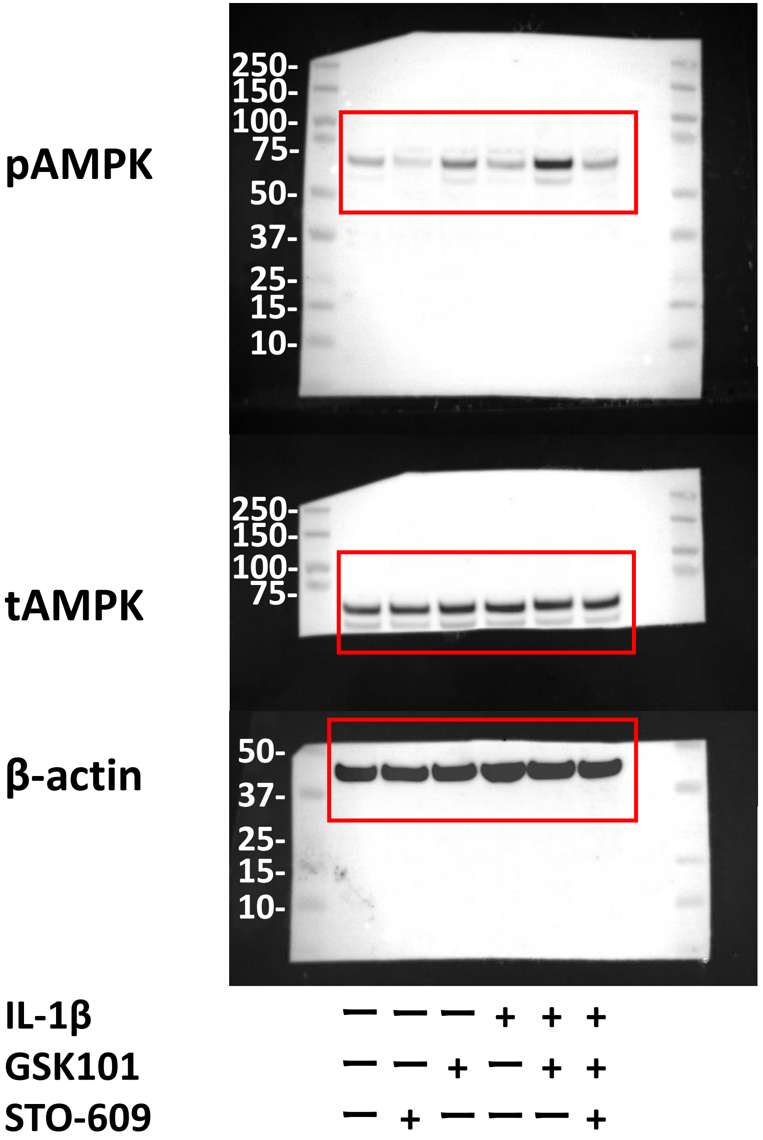


**Expression of pNFκB, tNFκB and β-actin in BACs in the Figure 3b**

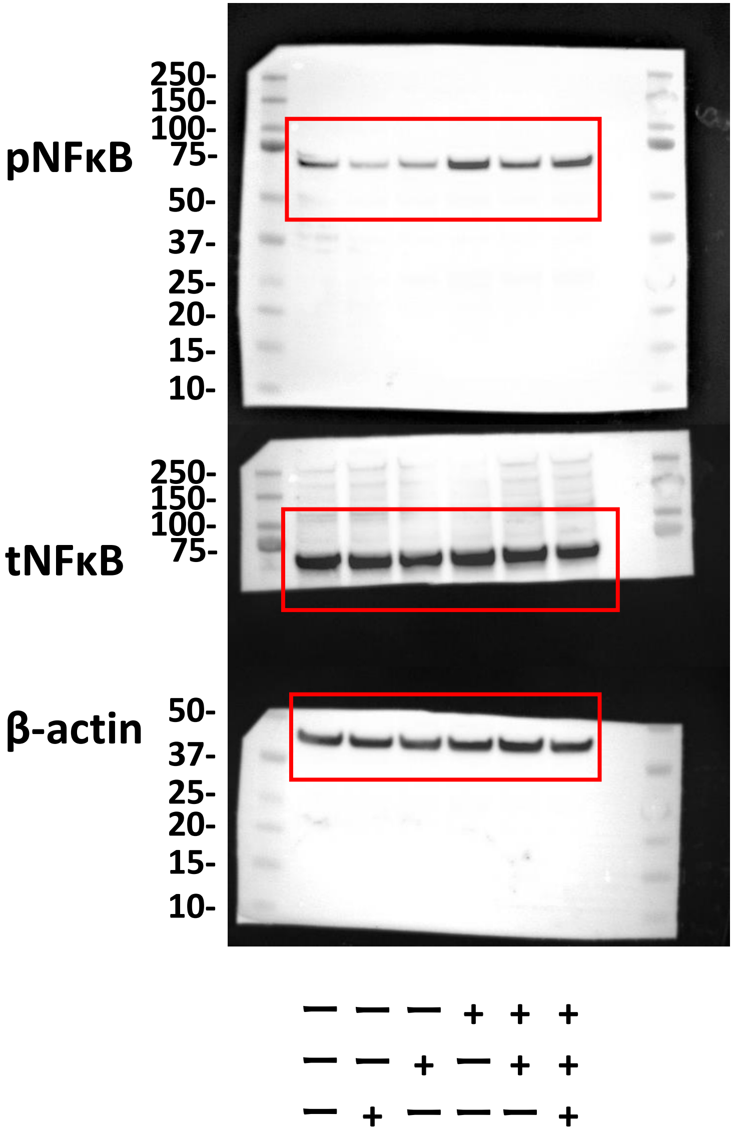

# Supplementary data 4

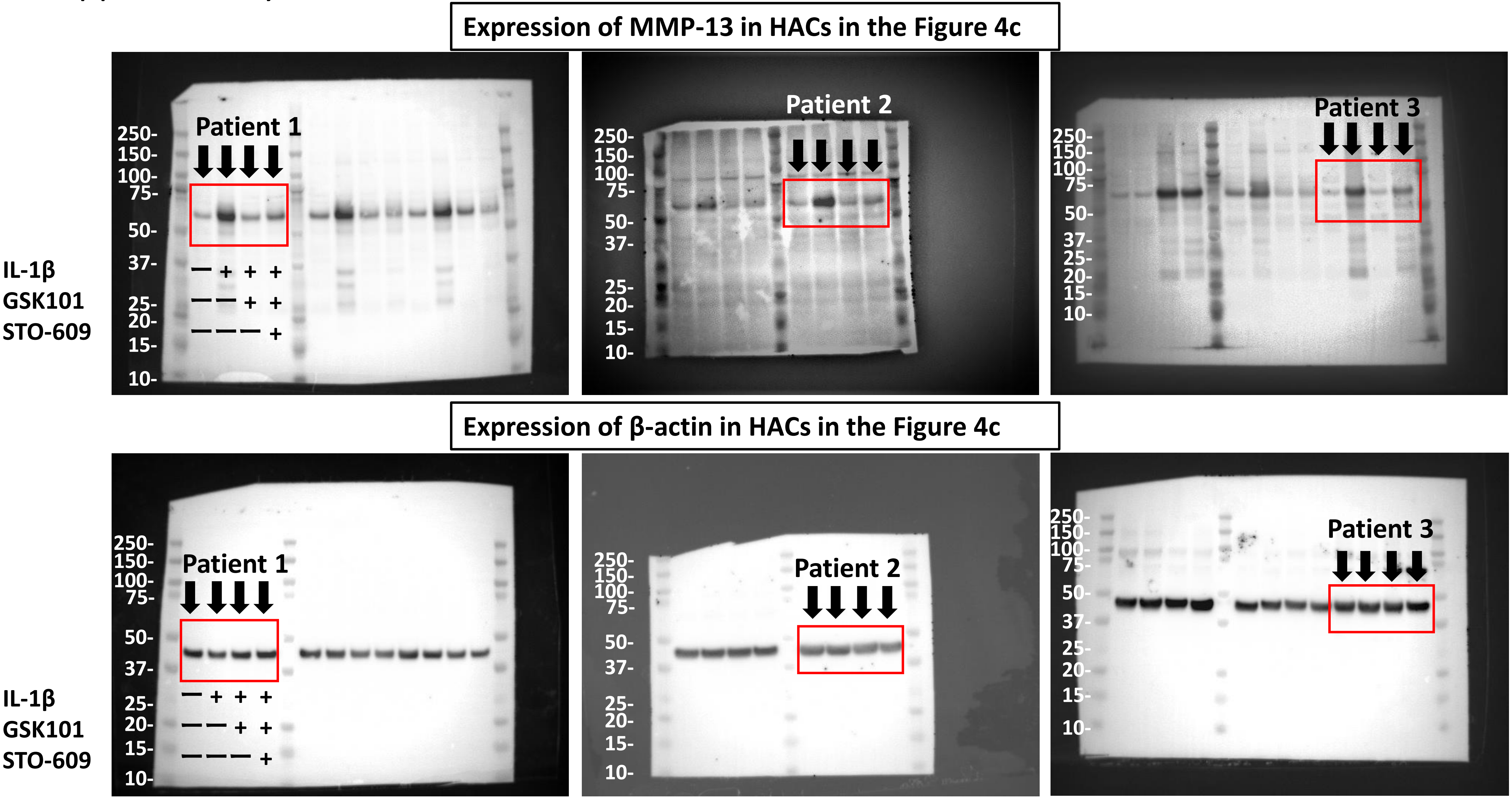

Supplementary data 1. Original membranes of MMP-13 and β-actin of Western blot analysis of Figure 2b in the manuscript using HACs from three patients.*Co-treated with STO-609. †Co-treated with other agents which were not effective. HAC: human articular chondrocyte.

Supplementary data 2. Three replicates of explant culture of bovine articular cartilage tissue in Safranin O/Fast Green staining on day 7 in each group in Figure 2d. A substantial amount of proteoglycans was lost from explants treated with IL-1β, while this effect was almost completely rescued by co-treatment with IL-1β and GSK101.

Supplementary data 3. Original membranes of pAMPK and pNFκB of Western blot analysis of Figure 3 in the manuscript using BACs treated with IL-1β, GSK101 and (a) Compound C and (b) STO-609. BAC: bovine articular chondrocyte; p: phospho-; t: total-.

Supplementary data 4. Original membranes of MMP-13 of Western blot analysis of Figure 4c in the manuscript using HACs from three patients. HAC: human articular chondrocyte.
